# Prediction and Decision-Making in Intelligent Environments Supported by Knowledge Graphs, A Systematic Review

**DOI:** 10.3390/s19081774

**Published:** 2019-04-13

**Authors:** Elvira Amador-Domínguez, Emilio Serrano, Daniel Manrique, Juan F. De Paz

**Affiliations:** 1 Ontology Engineering Group, Department of Artificial Intelligence, ETSI Informáticos, Universidad Politécnica de Madrid, 28660 Madrid, Spain; eamador@fi.upm.es; 2 Artificial Intelligence Lab, Department of Artificial Intelligence, ETSI Informáticos, Universidad Politécnica de Madrid, 28660 Madrid, Spain; dmanrique@fi.upm.es; 3 Expert Systems and Applications Lab, Faculty of Science, University of Salamanca, 37007 Salamanca, Spain; fcofds@usal.es

**Keywords:** knowledge base, knowledge graph, intelligent environment, ambient intelligence, reasoning model, knowledge graph embedding

## Abstract

Ambient Intelligence is currently a lively application domain of Artificial Intelligence and has become the central subject of multiple initiatives worldwide. Several approaches inside this domain make use of knowledge bases or knowledge graphs, both previously existing and ad hoc. This form of representation allows heterogeneous data gathered from diverse sources to be contextualized and combined to create relevant information for intelligent systems, usually following higher level constraints defined by an ontology. In this work, we conduct a systematic review of the existing usages of knowledge bases in intelligent environments, as well as an in-depth study of the predictive and decision-making models employed. Finally, we present a use case for smart homes and illustrate the use and advantages of Knowledge Graph Embeddings in this context.

## 1. Introduction

According to Augusto et al.: “An Intelligent Environment is one in which the actions of numerous networked controllers is orchestrated by self-programming pre-emptive processes in such a way as to create an interactive holistic functionality that enhances occupants experiences” [1]. Although in this particular context the term “*environment*” is popularly associated with homes, it also encompasses broader scenarios, such as buildings, streets or other areas. Intelligent environments are technologically based on the combination of several socio-technical innovations such as the Internet of Things (IoT), mobile Internet access, smartphones, data analytics, open data initiatives, and sharing economy models [2]. These advances allow intelligent environments to manage assets and resources efficiently by services enhanced with intelligence such as traffic management or healthcare systems.

Developing responsive and smarter environments is one of the main present objectives, as shown by the number of research projects developed to pursue this goal, such as Km4City [3] or RoomPathy [4]. Although there exists a considerable heterogeneity among the existing works in terms of objectives, methods, and areas of application, the use of knowledge bases (KBs) or knowledge graphs (KGs) is in the core of a large number of these works.

KBs play a key role in multiple ambient intelligence applications, as they are an essential part of the conversion of heterogeneous, numerical data provided by sensors into contextualized and semantic information. The transformation procedure is usually performed using ontologies, which enable the conversion of unstructured data into knowledge. The nature of this knowledge can be very diverse, depending on its source, its application domain, or its specific purpose. The use of KBs supports formal reasoning and the construction of machine learning models, both supervised and unsupervised.

This paper contributes with a systematic review of the main existing applications of KB across the different domains of application of ambient intelligence, studying which are the most common usages per area. Furthermore, we extract general features of the works existing within a particular area, as well as its main issues and shortcomings. In addition, we emphasize the different decision-making and prediction methods identified in the literature, evaluating them according to some of the most relevant and desirable features of them: scalability, interpretability, predictive capability and resource consumption.

As a result of this review, we identify a significant opportunity in the use of KB for intelligent environments: improving the trade-off between scalability and predictive capability. We also propose the use of Knowledge Graph Embeddings (KGE) in the particular case of smart homes. The use of this technology, which supports scalable predictions with uncertain information [5], is usually ignored by the specialized literature according to the systematic review presented here.

The remainder of the paper is structured as follows. After reviewing the research methodology in Section 2, Section 3 revises the usage of KB across several domains of application of ambient intelligence. Section 4 focuses on reasoning and prediction models applied in these works. Section 5 describes a use case to illustrate the use of KGE in smart homes. Finally, Section 6 draws the conclusions.

## 2. Research Methodology

**Figure 1:**
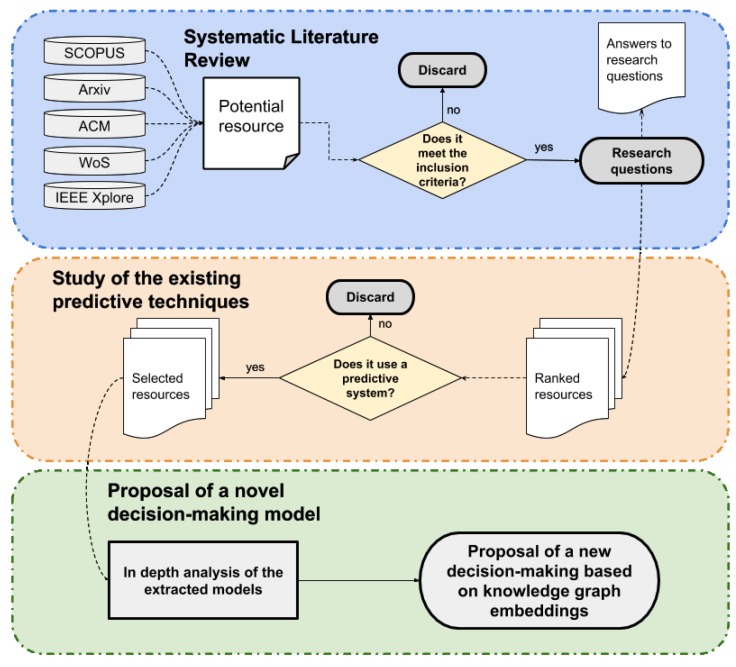
Schema of the workflow of the conducted study.

The guidelines to review software engineering have been adapted to conduct this review [6]. According to them, a systematic literature review comprises three main steps: *planning*, *conducting* and *reporting*. Furthermore, after the most relevant resources are identified, a more in-depth study about the employed decision-making and predictive techniques is presented. This study identifies the most used techniques, their advantages, and their flaws. Finally, an example of a reasoning system based on KGE applied to a smart home environment is presented. Figure 1 shows an overview of the entire paper contributions into its three different stages.

### 2.1. Planning

Regarding the research planning, the primary goal is to clearly define the protocol, parameters, and scope in which the review is performed. The main goal of the review has to be defined before starting to plan so that the protocol defined afterward is perfectly aligned with this objective. In this case, the goal is to *identify which are the fundamental current uses of knowledge bases in intelligent environments*. More specifically, we are interested in finding whether there are works where these KBs are exploited by a reasoning system to make predictions and decisions, as opposed to only serving as a source of information. It is also necessary to clarify that, in the given context, the term *“knowledge”* refers to meaningful, structured information interconnected in a network structure.

To achieve this goal, as well as providing a good overview of the current state-of-the-art in KBs applications for ambient intelligence, we propose the following research questions:

- How are knowledge bases for ambient intelligence generated?
- Which are the principal sources of data for knowledge base generation?
- Are ontologies involved in the creation procedure?
- For what purposes are knowledge bases used?
- What are the most used predictive techniques for knowledge bases in intelligent environments?
- Why have those techniques been selected?

Then, we have to establish a search strategy, which consists of two steps. The first step is to ascertain which are the main search terms, as well as the queries to search into the different sources to obtain the desired resources. The most important terms are *‘knowledge base’, ‘knowledge graph’* and *‘intelligent environment’*. However, the term *‘intelligent environment’* is too general, as more specific terms can be used to cover the different applications existing inside the field. To cover more relevant works, we have extended the search term list with concepts such as *‘smart waste’, ‘smart grid’, ‘smart lighting’, ‘smart homes’, ‘digital city’* and *‘smart city’*. Equivalent terms such as *‘ambient intelligence’* and *‘situational awareness’* are also included. In addition, to parallelize the systematic query process, we have made use of the Boolean operators (AND, OR) available in most of the existing online databases. All generated queries follow the pattern: *(“knowledge base” OR “knowledge graph”) AND **“{search term}”***.

Once we have defined the set of terms to look up, the following step is to determine which are the sources to use. In this work, we have selected five digital databases: SCOPUS, ACM, Arxiv, Web of Science and IEEE Xplore. Table 1 presents the relation of total and selected resources per database.

Table 2 describes the inclusion and exclusion criteria adopted to determine which of the obtained results are valid for the review. Inclusion clauses are conjunctive, whereas the exclusion ones are disjunctive. Among the rejection criteria, the works published before 2012 are discarded. The motivation is that the term *“knowledge graph”*, as we conceive it nowadays, was not coined until 2012 [7]. Therefore, all the papers employing this term to refer to KB organized in the structure of a graph date after this year.

Moreover, deep learning techniques started to have a hegemonic position among the machine learning methods also during this year (In the ILSVRC-2012 competition on ImageNet, Convolutional Neural Networks achieved accuracies never seen before in computer vision). As stated, this review is particularly interested in the use of state of the art in machine learning and Natural Language Processing (NLP) techniques with predictive purposes in intelligent environments. In those cases where there exists a set of separate papers that are part of the same project, we consider them as a whole, referencing them by the name of the project they are involved.

### 2.2. Conducting

Considering the sparsity of the search terms, which leads to a considerable heterogeneity amongst the resulting works, we rank the extracted papers according to their correlation with the proposed objective as in Fernandez et al. [8] and Khan et al. [9]. A rank between 0 and 5 is assigned to each work to assess the relevance of each paper concerning the subject of the study, being 5 *“extremely relevant”* and 0 *“completely irrelevant”*. Five evaluation criteria are considered to evaluate the relevance of each resource, namely:

- Clear explanation of the use of knowledge bases.
- Ontology use for knowledge base generation.
- Definition of the application domain.
- Use of decision-making or predictive models.
- Use of machine learning techniques for predictive purposes.

For each criterion, three different values can be given depending on the degree of fulfillment the paper provides: *“completely fulfilled"*, *“partially fulfilled"* and *“unfulfilled"*. Each of these values is associated with the numerals 1, 0.5 and 0, respectively. Once the criteria have been evaluated for a resource, its final relevance factor is the sum of all the scores given for each criterion.

This classification also serves as a filter for the following parts of the review where the reasoning techniques are studied more in depth. We only consider those works that achieve a score equal or higher than 2.5.

## 3. Ambient Intelligence Applications on Particular Domains

Ambient intelligence is nowadays effectively applied, although there are works that date before 2003. The development of smarter sensors, as well as the implementation of these sensors in conventional devices, such as smartphones, has made it possible to collect a considerable amount of data to support intelligence services. Aside from the technological aspect, another crucial factor in this growth is the mass use of social media, becoming one of the most important information sources. Although this data is not obtained directly by sensors but provided by the own citizens, it is equally relevant for the development of intelligent environments, as noted in Goodchild [10].

On the other hand, this heterogeneous data collected by sensors lacks context and semantics, hindering its use for automatic reasoning. KBs are a mainstream technology to provide context for this data, make it readable, and enable human-like reasoning over it. These bases not only can represent what is called *common-sense knowledge*, which is the kind of reasoning that is innate to humans, but also can be used to synthesize the numerical heterogeneous data provided by sensors as information or facts, and combine these with the knowledge obtained from different sources, such as texts or social media posts.

Tandon et al. [11] provide an insight on the multiple uses of commonsense knowledge for machine learning. Furthermore, one of the uses presented in their work explicitly concerns smart vehicles and the difficulties that they face in situations whose solutions are innate to humans. The introduction of commonsense knowledge could potentially enhance the decision-making process, as it would increase the contextual information available, which would lead to better responses. WordNet [12] and Cyc [13] were some of the firstly developed commonsense knowledge bases. Whereas the nature of the information contained in WordNet is mostly semantic, Cyc is, according to their authors: “a formalized representation of a vast quantity of fundamental human knowledge: facts, rules of thumb, and heuristics for reasoning about the objects and events of everyday life”. WordNet is to this day still one of the most used KBs, as it enables entity mapping across different KBs. This is the case of WebChild [14], an open access and updated version of Cyc, whose entries are directly mapped to their matching ones in WordNet, easing their access. WebChild contains not only measurable and demonstrable facts but also abstract information, such as opinions or sensations, which can be particularly useful in the context of intelligent environments, such as smart homes. Finally, ConceptNet [15] emerges as a multilingual semantic knowledge graph, overcoming the main problem existing in WordNet: its particular limitation to English. Its use has been successfully tested on chatbots, enabling their understanding and generation of human-like responses.

**Figure 2:**
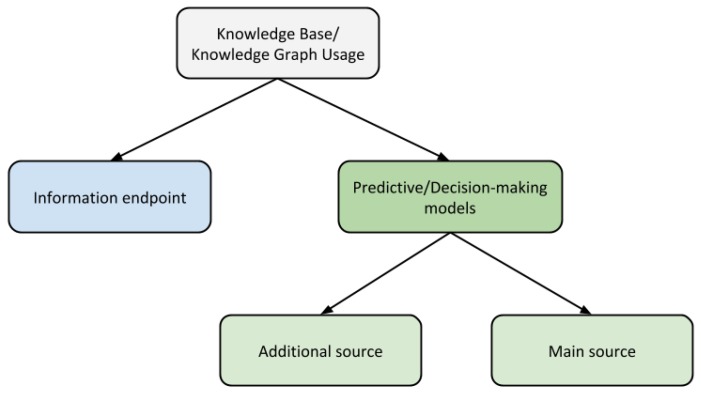
Classification of the main applications of knowledge bases on intelligent environments.

Nonetheless, the usage of KBs in intelligent environments changes qualitatively across the different existing domains of application. Before a more conscious study, it is necessary to provide a taxonomy of the identified applications of KBs. Figure 2 presents the proposed taxonomy, composed of two disjoint categories and a more specific distinction for one of them. According to this classification, we can distinguish two main applications:

- As an endpoint: In this approach, the KB serves only as a source of information to consult, and conducts no further reasoning over this data.
- As support for the predictive system: In other cases, the information provided by KBs is used by a decision-making system to perform different tasks, such as making recommendations or predictions. Inside these approaches, we distinguish two different kinds: systems where the knowledge base is the principal or only source of information, and those where it is an additional or auxiliary source. In this second type, the predictions are based on heterogeneous data, and the KB serves only as a support for contextual information that is not necessarily employed.

Table 3 presents the selected resources as well as the itemization of the quality assessment for each of them.

**Figure 3:**
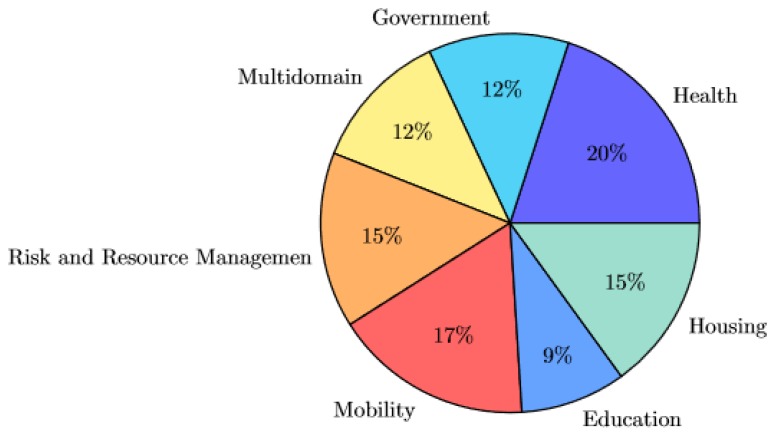
Pie chart representing the proportion of papers per identified domain.

Once the selection process is finished, we proceed to classify the resulting resources according to their application domain. In this case, we have separated the considered works into seven different categories, being: *health*, *housing*, *mobility*, *risk and resource management*, *education* and *government*. This taxonomy is roughly based on the one presented by Ahmed et al. [51], with the addition of the categories *“government"* and *“education"*. Those works where the exact application domain is not specified are classified as *multidomain*. Although the presented categories are disjoint, some of the selected resources could be suitable for more than one category. This is particularly noticeable in the case of housing and health domains, as some of the works inside the housing domain are related to health monitorization and eldercare. Nonetheless, aside from this particular case, no other overlaps have been found. Classification is performed according to the specific application presented by the authors. Figure 3 reflects the proportion of resources classified per domain.

### 3.1. Health

Providing smarter healthcare solutions, usually referred to as *e-health*, is one of the main concerns of ambient intelligence, as shown by the number of works dedicated to this particular issue. In the majority of the considered resources, KBs serve as the primary source of information for diagnosing systems. Rule-based expert systems are the most prolific in this domain due to its specialized nature, as they offer high interpretability and possess the capability of modeling uncertainty. In some of the selected resources [22,40,47], the fuzzy predictive rules of the system are designed manually by experts.

In Kim and Chung [22], an estimation of the depression index of the population is obtained from crowdsourced information, in combination with the contextual information of the individuals and the knowledge provided by experts. The fuzzy model presented by Ali and Lee [40] focuses on providing wellbeing recommendations to users, whereas the approach proposed by Martín-Ruiz et al. [47] aims to monitor children’s neurodevelopment in an intelligent environment for early diagnosis.

Other approaches, such as Peral et al. [35] or Riboni et al. [50], rely on machine learning techniques for generating predictive models, proposing a classification tree and a random forest, respectively. These approaches allow diagnosing models to be automatically inferred from the data, improving the scalability of the previously presented works, while still maintaining a deep level of interpretability.

Other works do not focus on diagnosing but on patient assistance and particularly eldercare [27,42]. In these works, the goal is not to detect whether the patient suffers or not from a specific disease, but to monitor and help them to conduct everyday tasks. For this purpose, ambient intelligence context-modeling is conducted, generating a clear and accurate overview of the environmental context of the patient, as to detect any potential anomalies in their behavior.

The most common technique for KB generation on this field is combining the data collected by sensors with the information provided by the medical reports of the patients. It is usual that differently specific ontologies transform the data into contextualized information. Another distinctive feature is that most of these works provide software that is available to users, but KBs and collected data are typically private and not available for other researchers.

### 3.2. Mobility

Transportation and urban mobility is another main category of ambient intelligence applications that leverage KBs. As opposed to the health domain, where most of the presented works followed similar guidelines and characteristics, there is considerable heterogeneity in this field. Among the identified works, the most relevant is the project Sii-Mobility [18]. This project encompasses several papers presenting applications of KBs for different transportation issues. For instance, in Bellini et al. [17], a tool capable of extracting information via SPARQL queries from several existing KB endpoints is presented. The developed tool has been not only successfully used in Sii-Mobility for providing traffic-related information to users, but also for several other projects such as ECLAP [52]. In Badii et al. [16], the information extracted from the Sii-Mobility KB, as well as the data provided by sensors, serves as an input for a Bayesian Regularization Neural Network capable of predicting available parking spots in garages.

Except for 3cixty [19], which presents a tool for consulting a tourism and transport knowledge graph, the rest of the selected papers in this review use KBs for predictive purposes. Giannakopoulou et al. [24], Olszewski and Turek [26] and Zavala et al. [30] combine the KBs with data extracted from sensors and citizens, either from social media or by employing gamification techniques. Then, they combine this information for different purposes, namely predicting the best route available, diagnosing spatial problems on specific areas and context-modelling for smart vehicles, respectively.

Opposite to the previously presented works, where the selected predictive models were based either on rules or heuristics, Persaud et al. [20] propose a deep learning model for self-driven vehicles where predictions are performed based on a combination of images, sensor data, and the information gathered in a commonsense KB that serves as a support. The KB presented in this work is obtained directly from WebChild [14], and it has been successfully evaluated on reduced-scale experiments.

### 3.3. Risk and Resource Management

Works presented in this category are mainly aimed at risk prevention, addressing services such as city surveillance [25], resource management [28], or emergency simulation [32]. In some of them, KBs play a secondary role, serving as support for the predictive models as in the works by Orlowski et al. [25], Barnwal et al. [28] or Xu and Li [32]. These systems present decision support systems that, for the application domain, have to be supervised by humans. Therefore, they are typically based on rule-based techniques and probabilistic distributions. They generate the presented KBs by combining existing data sources, with data provided by users via social media, and data collected by sensors subsequently contextualized into information using ontologies.

Roffia et al. [34] present tools for querying the created KBs, as well as sending notifications to users when certain events happen. These KBs only work as a persistent layer. The approach presented by Sermet and Demir [48] is particularly interesting, as it presents a flood emergency system that includes a module based on NLP that is capable of interacting with the user in a question-answer fashion. This system retrieves the requested information from the KB according to the user’s input in natural language and responds accordingly. It enables not only notifications when flooding occurs, as in Barnwal et al. [28], but also real-time queries on the state of the system.

Unlike in the health domain, generated data and tools are publicly available in the revised works.

### 3.4. Housing

Smart homes are one of the main domains of application of ambient intelligence. Context-modelling is among the significant issues regarding intelligent environments, which in the housing domain is strongly associated with task recognition. Task recognition poses several challenges, such as user preference detection or component-dependency detection. In Rhee et al. [46], a rule-based system is presented to infer the user’s preferences from their context to enhance an assistant system, whereas Dimitrov et al. [45] use a Bayesian Network to model the dependencies between components to assist monitorization and error detection.

In this field, data are collected by sensors and contextualized employing different ontologies to create a KB. Vision systems are also employed frequently to retrieve information. Over the generated KB, several predictive techniques can be applied that fit the existing issue. For example, Machado et al. [44] introduce an approach for task recognition based on Bayesian Networks, whereas Hao et al. [41] rely on Formal Concept Analysis and vector similarity.

Particularly remarkable is the work proposed by Fast et al. [49], who present a novel approach for task recognition. In this approach, they generate a KB from publicly available stories published by users, using NLP techniques to detect which objects and actions are related and how. To do so, they define a set of actions that comprise a KB. From an image, the presented tool is capable of detecting the appearing components. Then, using the previously stored information, their tool identifies the occurring action by comparing the pattern of objects and actions presented with the ones allocated in the knowledge base.

### 3.5. Government

Smart government is also one of the most growing and promising domains in ambient intelligence. With the increment of citizen participation and open governments, many new available data have been generated to be formalized by KBs, which then can be used to query them or for prediction making.

Olszewski et al. [21] present a model for urban revitalization, in which they develop a KB from open and crowdsourced user data. From the generated KB, a fuzzy logic rule system is proposed to detect the main issues existing in the urban environment, as well as the preferences of the citizens.

However, most of the works considered aim for citizen query [27,31,38]. In Santos et al. [38], a city KG is generated such as dashboards can be automatically built upon to monitor the urban environment. Chung et al. [27] present an assistant system for distributing citizen complaints to their adequate city departments.

A remarkable particularity of these works is that, unlike the rest of the domains revised in this paper, the fundamental source of information is not sensors but crowdsourced data willingly provided by citizens.

### 3.6. Education

Only three works in the education domain have been considered relevant in this systematic review. Therefore, this hinders us from obtaining general conclusions. For instance, the work presented by Xiaobo et al. [23] can be classified under the querying branch of the taxonomy presented in Figure 2, as they provide a method for creating a KB for languages teaching that users can query, but no further operations are performed. On the other hand, other works [33,39] provide a first-order logic decision-making model, as well as the KB creation methodology.

Aguilar et al. [39] perform context-modeling to enhance the educational experience of the students, by taking context-dependant decisions that trigger specific actions both in physical (smart blackboards, smart desks) and virtual actuators (software components). Duyen and Nhon [33] present an intelligent guiding system for geometry teaching.

As in the government domain, data rarely come from sensors in these works but from existing KBs, experts, or text resources.

### 3.7. Multidomain

Some of the relevant works found in this review provide general solutions that can be adapted to some application domains. These papers provide approaches for KB generation that can be subsequently used both for querying and generating predictive models. Qiu et al. [36], Shan and Cao [37], and Schoonenberg and Farid [53] present frameworks for creating urban knowledge graphs, where all the components within the environment and the relations between them are represented. To generate these KGs, they employ different techniques. Qiu et al. [36] use deep learning techniques, such as Word2Vec [54] to extract the relevant existing entities and the relations that connect them from textual and sensor-collected data. Shan and Cao [37], and Schoonenberg and Farid [53] formalize KGs in a matrix-like form where different transformation operations can be performed to homogenize the data.

Bogale et al. [29] not only propose a KG generation approach, but also discuss the use of machine learning techniques over KGs for different tasks, such as data acquisition or knowledge discovery.

Since works under this category do not cover a specific domain, they do not use ontologies to convert the data unlike most of the previously presented works.

### 3.8. General Aspects of Knowledge Base Development for Intelligent Environments

As stated in Section 2, one of the goals of the presented review is to extract what are the most common practices on knowledge base creation for intelligent environments. From a simplistic point of view, we can distinguish between two main stages: data generation and contextualization.

Regarding data generation, sensors are the primary source of information. This affirmation is expected given the present domain, as sensors are on the foundation base of intelligent environments. However, it is remarkable that citizens also play a significant role in data generation, providing information either in a passive way, as in the case of medical reports, or in an active way. Citizen participation is particularly significant in the *government* and *education* domains, where data provided by citizens severely outsizes the amount of data provided by sensors. In addition, in these areas, there is a tendency to reusing existing open KBs.

Commonsense KBs play an essential role in the development of ambient intelligence systems. This affirmation is made evident in works such as Peral et al. [35] or Fast et al. [49], where WordNet is used to enhance the outputs of the decision-making system in the first case, and for data contextualization in the latter. The presence of commonsense KBs is particularly noticeable in Persaud et al. [20], where the developed KB for a self-driven vehicle is primarily founded on the information related to mobility extracted from WebChild.

**Figure 4:**
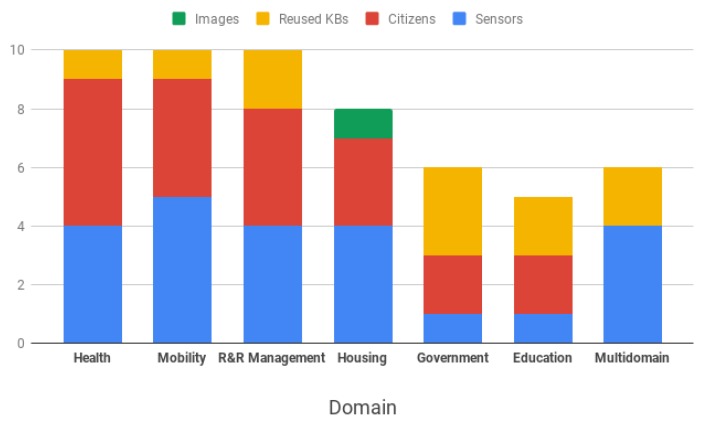
Proportion of data per source across the studied domains according to the studied resources.

Citizen data also have a considerable influence in other areas, such as risk and resource management or mobility, where data are extracted mostly from social media platforms. An example of knowledge extraction from social media is illustrated in Puri et al. [55], which presents an algorithm for linking published tweets to existing ordinances. This linkage is done employing commonsense KBs, and each tweet is associated with an existing ordinance, subsequently creating new information stored in the KB. Figure 4 depicts the proportion of data per source on the presented domains.

Regarding contextualization, different domain ontologies are employed to generate the corresponding KBs. However, not all data gathered is contextualized, as in those works using KBs as a support for the predictive system. The information contained in the KB is mostly general and static over time. However, the study of the most used ontologies for intelligent environments is out of the scope of this work, although Refs. [56,57,58] reference different reviews on the issue.

## 4. Decision-Making and Predictive Models in Intelligent Environments

After revising the main application domains of the relevant works found in the literature, this section details the decision-making and predictive techniques employed in the studied works. An assessment of the advantages and disadvantages of each of the proposed predictive models is also presented to identify which are the most used techniques and why. For this purpose, we evaluate each of the presented techniques according to their scalability, their interpretability, their predictive capability, and their resource consumption. The importance of the KB on the decision-making or prediction process is also considered. Following similar criteria as the ones used to rank the relevance, we give a score according to the features mentioned earlier.

As shown in Table 4, there is considerable heterogeneity among the employed methods, although most of them present a common and desirable feature: they are interpretable. Olszewski and Turek [26] train a neural network that outperforms the accuracy of decision tree based models such as CART. However, the authors finally decide on the CART model because of its superior interpretability assuming a worse predictive power. This decision is significant, as there is a conscious loss on accuracy for the benefit of obtaining models that can be understandable by humans, and where decisions can be explained by means of their inputs.

Following this trend, we find that there are several works where the selected methods present a high degree of interpretability, such as fuzzy rules [65], RIPPER rules [71] or C4.5 trees [66]. Interpretability is particularly noticeable in those fields that require human interaction and decision, like *health* and *risk and resource management*, and less strongly in *mobility*. However, the selected methods for these areas show a considerable trade-off between their degree of interpretability and their scalability and predictive power. Random forests [67] and CART trees [64] offer one of the best scalability, prediction power, and interpretability relation, making them useful and suitable techniques for the different application domains.

Under these interpretable predictions techniques, several methods out of the scope of machine learning appear in the relevant works. Experts usually develop these models ad hoc, not requiring a training phase. In this vein, Hao et al. [41] employ a heuristic based algorithm. Duyen and Nhon [33] utilize a set of if-then clauses which are generated based on the information existing in the KB. The rule sets developed by experts is the most common non-machine-learning method as shown in Table 4. As referred in Section 3, rule-based systems are particularly widespread in the health domain, where there exists a tradition of the use of human-developed expert systems as Mycin [72].

In contrast to the rule-oriented approaches, we observe that there are two main trends: models based on Bayesian networks, and models based on artificial neural networks, as well as existing combinations of these two learning paradigms, Bayesian Regression Artificial Neural Networks, or BRANN [68,69,70]. This model, used in the Sii-Mobility project [16], is well suited for working with KBs and for the mobility domain. This is because the model has not only high predictive power but also mechanisms to explain its predictions although in a less intuitive manner than trees or rules. The main benefit of Bayesian networks is that they are capable of working with uncertainty, as they are based on probabilistic distributions. Moreover, expert knowledge about the conditional dependencies of the different variables can be easily integrated into these models. However, their performance and required resources are directly related to the characteristics of the input data. The higher the number of features to represent, the more the number of nodes the Bayesian network needs, making the computational power required and the complexity of the network increase proportionally.

Neural networks offer a mathematical model that computes the output by nonlinear transformations of the weighted inputs associated with each neuron. The main advantage of such models is their predictive power since they can solve much more complex problems than rule-based and statistical methods. These networks not only can generalize the training data but also predict unseen or infrequent inputs. The computational power required is directly proportional to their size, which, unlike Bayesian networks, is defined independently of the number of features, except in the input layer. However, since more complex neural networks can solve more difficult problems, deep neural networks come into play with a large number of neurons in each layer (Persaud et al. [20] and Chung et al. [43]). Nonetheless, neural networks present the significant disadvantage of being “black box” classifiers, since they cannot explain the predictions in terms of the input. As mentioned earlier in this section, interpretability is one of the most desirable characteristics of decision-making models for ambient intelligence, leading to restricted use of these approaches.

Fast et al. [49] combine neural networks with explainable techniques for task recognition in smart homes. This work presents a three-step procedure, where vectors for each activity and each object associated with every activity are generated using Word2Vec [54]. Then, the prediction is based on the similarity of the word vectors describing the captured image with the ones existing in the KB for that activity. However, the construction of this vector space is expensive in terms of resources, as about 1.8 billion words are used to train the model and an incremental approach is followed, where activity vectors and object vectors are constructed separately and then joined. The vector generating procedure is based on the frequency and the co-occurrence of words in a text corpus, similar to a bag of words approach.

Although this approach deals with the interpretability, the generating procedure of the vectors does not ensure capturing fine-grained information such as co-dependencies existing between actions or objects. Another shortcoming of the approach is its poor scalability, requiring to retrain the whole model if new objects or new actions are presented. A potential solution for this problem is the use of knowledge graph embedding techniques (KGEs), which generate a vector for each entity and, depending on the approach, each relation, based on the information collected in a KG. An essential feature of KGEs is that it preserves the similarity between entities so that elements that are similar and related to the same entities are close in the generated vector space.

Current KGE models have been proven to be scalable, easy to train, and competitive in performance [5,73]. To train these models, a KB or KG is needed, that can be either an existing one, ad hoc for the system, or a combination of both. The use of an ontology over the triples can support tasks such as enhancing the KB with inferable information. For example, considering the entity *Barack Obama* as an instance of a class *Person*, it follows that the entity has to have a gender, age or height, even when the KB may not include this information.

This representation is suitable for several tasks such as question answering or recommendation systems, which Section 3 presents as existing issues in ambient intelligence. The following section describes an approach based on KGE for solving different issues in the smart home domain.

## 5. Use Case: Knowledge Graph Embeddings for Smart Homes

A KG is a specific formalization of a KB where the objects or entities are represented as nodes in a graph, and the edges indicate existing relations between them. A KB codified in Resource Description Framework [74] (RDF) triples is essentially a KG where a *fact* presents the existing relationship between two entities in the format (subject,relation,object). KGs are suitable for representing information about a single domain or several of them. In some of the works reviewed in Section 3 [36,53], KGs were used to cover a global domain.

KGs serve as the base for multiple applications, virtual assistants being one of the most popular ones. For example, Google Assistant, which is one of the most used assistants is built over the Google Knowledge Graph [7]. This KG aims to represent knowledge across a universal domain. On a much more reduced scale, we present an example of KG used in an intelligent environment, more precisely in smart homes. As mentioned in Section 4, there are domains where predictions have to be entirely understandable and supervised by humans, as in the case of health or mobility. However, this feature is not essential in the case of some smart homes’ applications, such as the ones to provide comfort, where potential failures could represent a much lower risk than in other areas.

From a general point of view, KGE techniques present the following benefits:

- Good trade-off between scalability and predictive capability.
- Capacity for dealing with uncertainty and incompleteness while still obtaining accurate predictions.
- The generated embeddings can be used by sub-symbolic predictive models, such as neural networks, to perform supervised classification.
- Easy use for high-level tasks as recommendation [75,76] and question-answering [77]. These two tasks are particularly relevant in the context of smart homes.

**Figure 5:**
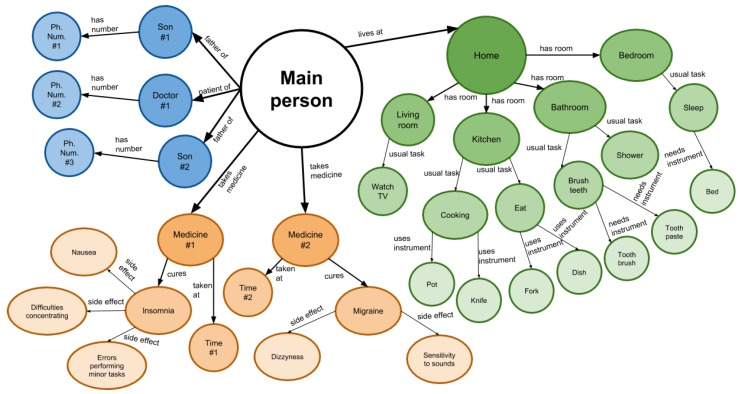
Example of Knowledge Graph for eldercare. The entities referring to humans are depicted in blue, to health in orange, and to housing in green.

In this example, we focus on the application of KGE for the development of an eldercare-oriented smart home. These systems are very important for our society because the amount of older adults who live alone is increasing and these environments can monitor their activities while still providing help and comfort. Figure 5 presents an example of a simple knowledge graph in the smart home domain for eldercare. The presented use case remains purely theoretical, as there has not been a physical implementation nor testing. Therefore, its role is to illustrate the benefits and the versatility of the use of KGE in intelligent environments.

To illustrate some of the tasks that a KGE built over a KG can solve, we present the following scenario based on the idea presented by Fast et al. [49]:

*Consider a house, composed of four separate rooms, each one provided with cameras. When a person enters the room, the cameras capture the scene, which is subsequently processed by a computer vision system that detects the objects that are currently being used by the person. The output of the system is the list of objects being used. The house also has sensors that capture information such as temperature and humidity.*

Apart from these external systems, we consider embeddings generated for each of the entities presented on the graph from Figure 5 using TransE [78]. TransE is a knowledge graph embedding technique that, although having low complexity in terms of parameters, has reached state-of-the-art performance in several tasks such as link prediction [79]. Due to its low complexity, it is easy to train and can scale up to very large KGs. Once the model has been trained, it obtains a set of entity embeddings E and a set of relation embeddings R. Other alternatives include RESCAL [80] or the Neural Tensor Networks (NTN) [81]. Some approaches enhance word vectors with Wikipedia information [82], which can also be considered for the presented problem.

### 5.1. Task Recognition

Task recognition is one of the main problems inside the smart home domain, as made evident by the works presented in Section 3. The action that is being conducted by the senior citizen could be predicted by using the previously calculated embeddings in combination with the output produced by the computer vision system. Since task recognition is very environment biased in some cases, we benefit from this fact to allocate specific actions under its corresponding rooms. For example, the action *“shower”* always takes place in the bathroom, whereas the action *“watch TV”* likely takes place in the living room. In this example, we present the predictive procedure for the action *“cooking”*.

Firstly, the computer vision system arranges a list of objects after processing an image captured by the kitchen camera. This list will contain objects such as *“pot”, “pan”* or *“knife”*. As we are trying to detect an action, we refer to the relation *“uses instrument”*, since this is the one we employed to represent the objects that are used by a person during a certain action. The relation *“needs instrument”* can also be considered.

Three elements compose a fact: a subject, a relation, and an object. However, in this case, two out of the three elements are known, which leads to different half-constructed facts with the form *(?, uses instrument, [pot, knife, pan, ...])*. Thus, the goal is to predict the missing element of the fact, which is, in this example, the action. This problem is known as triple or link prediction, and KGEs are a natural representation to solve it [5].

From the existing entity embeddings E and relation embeddings R, we can predict, for each incomplete fact, which is the most suitable subject. For this purpose, the subject of an incomplete fact is substituted by every potential and feasible entity. Once the triple is constructed, the model provides a score that represents its feasibility. As we know that the action is developing in the kitchen, the potential subjects are bounded to the actions associated with this area. If no action bounded to this area is detected, actions existing in other areas will be evaluated. The model calculates the feasibility of a fact by measuring the distance between the combination of the embeddings of the entities and the embedding of the relation. Once every potential fact has been evaluated, the triple with the best score is considered as the most suitable. In this case, the commonly predicted subject for all the previously half-constructed facts is *“cooking”*.

### 5.2. Symptom Detection

Now, we focus on the information provided both by the computer vision systems and the sensors to detect anomalies in the behavior patterns that could be a symptom of a disease. As presented in the previous task, some actions are very room bounded, and the objects associated with each action are usually constant. In the previous section, the goal was to predict, given the objects, which action was taking place. This task raises the opposite problem: detecting whether or not the instruments used are appropriate for a known action.

Figure 5 represents (orange nodes) that if a person suffers from migraine and insomnia, then he or she may have difficulty in concentrating and make errors performing minor tasks. Therefore, the proposed system can detect if these symptoms are potentially manifesting, and consequently notify his or her family or doctor. The procedure to measure the feasibility of each action-object pair remains the same as in the previous part: we evaluate each *(action, [uses instrument/needs instrument], object)* fact and obtain a value that represents its feasibility. If the credibility of an existing action-object pair reaches critically low values on a repetitive basis, then there exists a potential issue, which is automatically notified to the corresponding carer.

### 5.3. Personal Assistance

One of the main usages of KGE is to serve as a base for virtual assistance technologies. This application is particularly crucial in eldercare, as cognitive and memorizing capabilities tend to decrease with age. These difficulties hinders these people from remembering essential events, such as taking medicines at a particular time. A KG could serve as a storing place for all these reminders, as well as any other relevant information.

However, knowledge graphs can also be useful for guiding activities, as well as acting as recommendation systems. In the previous section, we exposed an approach to detect symptoms based on the disparity of an action-object pair. However, a disparity is not necessarily a symptom of a more worrisome issue. In some cases, the user just forgets which object to use in a precise moment, which can be avoided by assisting the person conducting the task.

For example, according to Figure 5, the task *“brush teeth”* is composed of two objects: *“toothbrush”* and *“toothpaste”*. This task links to the facts *(brush teeth, needs instrument, toothbrush)* and *(brush teeth, needs instrument, toothpaste)*. The relation *needs instrument*, unlike *uses instrument*, represents a bidirectional restriction between the action and the object, where the object is only associated with that task, and the task necessarily requires the object.

In this case, the action *“brush teeth”* strictly specifies the need of a toothbrush and toothpaste. Therefore, if the system detects that the user is using only one of these objects, then it can infer which are the remaining ones by using the triple prediction approach presented in the previous section. Once the system identifies the missing elements, they are communicated to the person to help them following with the current task.

This use case and the three issues considered in smart homes, and particularly eldercare, illustrates the possible use of KGEs in intelligent environments. The use of this technology, which supports scalable predictions with uncertain information [5], is ignored in the specialized literature according to the systematic review presented here.

## 6. Conclusions

We present in this work a review of the most relevant works regarding knowledge base (KB) use in intelligent environments, identifying two main possibilities: as an endpoint for queries or as a base for predictive models. Furthermore, we have studied each work in the context of the particular area they apply to, extracting relevant features for all the identified subdomains: health, mobility, education, government, housing, risk and resource management, and multidomain.

The conducted review suggests that:

- Most of the identified applications of KBs to intelligent environments are related to the health domain, closely followed by mobility.
- The primary source of data for KB development for intelligent environments comes from sensors and citizens. In some fields, such as government or education, there is a tendency of reusability of existing KBs.
- Rule-based approaches are the most proliferate decision-making models in ambient intelligence, particularly in works related to health and risk and resource management.
- The majority of the presented models are characterized by high interpretability and low resource consumption, although they present some scalability limitations.

From the performed study, we conclude that the use of Knowledge Graph Embeddings (KGEs) is mostly overlooked in the specialized literature of Intelligent Environments. This technology is capable of giving scalable predictions with uncertain information while they leverage naturally the information embedded in a KB, which is the core of most works in Intelligent Environments. A use case is presented in this paper to illustrate the use of KGEs in a smart home scenario, supporting issues such as task recognition, symptom detection, and personal assistance.

## Figures and Tables

**Table 1:** Relation of total and selected results for each source.

Source	Total Results	Selected Resources
SCOPUS	149	21
Arxiv	49	4
ACM	12	0
Web of Science	36	1
IEEE Xplore	87	8
**TOTAL**	**333**	**34**

**Table 2:** Established acceptance and rejection criteria.

Inclusion	Exclusion
Papers written in English	Papers not written in English
Papers that provide a clear insight into the usage of the knowledge base	Works published before 2012
Works that present a methodology for knowledge base generation in the given context	Papers that include one of the search terms amongst its keywords but do not make any further reference to them

**Table 3:** Quality assessment for each considered resource. The criteria is ranked as follows: X = 1 point, / = 0.5 points, empty cell = 0 points. The relevance factor is the addition of the singular values. Resources with scores lower than 2.5 are not reported.

Resource	C1	C2	C3	C4	C5	Relevance Factor
Sii-Mobility [16,17,18]	X	X	X	X	X	**5**
3cixty [19]	X	X	X			**3**
Persaud et al. [20]	X		X	X	X	**4**
Olszewski et al. [21]	X		X	X		**3**
Kim and Chung [22]	X		X	X		**3**
Xiaobo et al. [23]	X	X	X			**3**
Giannakopoulou et al. [24]	X		X	X		**3**
Orłowski et al. [25]	X		X	X		**3**
Olszewski and Turek [26]	X		X	X		**3**
Chung et al. [27]	X	X	X	/		**3, 5**
Barnwal et al. [28]	X		X	X		**3**
Bogale et al. [29]	X	X		X	X	**4**
Zavala et al. [30]	X		X	X		**3**
Zhou et al. [31]	X	X	X			**3**
Xu and Li [32]	X		X	X		**3**
Duyen and Nhon [33]	X		X	X		**3**
Roffia et al. [34]	X	X	X			**3**
Peral et al. [35]	X	X	X	X		**4**
Qiu et al. [36]	X			X	X	**3**
Shan and Cao [37]	X			X	X	**3**
Santos et al. [38]	X	X	X			**3**
Schoonenberg and Farid [38]	X	X	X			**3**
Aguilar et al. [39]	X	X	X	/		**3, 5**
Ali and Lee [40]	X	X	X			**3**
Hao et al. [41]	X	X	X	X	/	**4, 5**
Kim and Chung [42]	X	X	X	X	X	**5**
Chung et al. [43]	X	X	X	X	X	**5**
Machado et al. [44]	X	X	X	X	X	**5**
Dimitrov et al. [45]	X	X	X	X	X	**5**
Rhee et al. [46]	X	X	X			**3**
Martín-Ruiz et al. [47]	X	X	X	X		**4**
Sermet and Demir [48]	X	X	X			**3**
Fast et al. [49]	X		X	X	X	**4**
Riboni et al. [50]	X	X	X	X	X	**5**

**Table 4:** Application domain and assessment of the works employing predictive techniques over knowledge bases. The symbolism is as follows: X = yes/high, / = partly/medium, empty = no/low.

Resource	Domain	Predictive Technique	Scalability	Interpretability	Abstraction Capability	Low Resource Consumption	Relevance of KB	Overall Score
**Ali and Lee** [40]	*Health*	*Rules Manually Created by Experts*		X		X	X	3
**Barnwal et al.** [28]	*Risk/Resource Management*	*Probabilistic distributions*		X		X	X	3
**Bogale et al.** [29]	*Multidomain*	*Multivariate Linear Regression*	/	/	/	X	X	3.5
**Chung et al.** [43]	*Health*	*Deep Neural Network*	X		X		X	3
**Dimitrov et al.** [45]	*Housing*	*Bayesian Network* [59]		X		X	X	3
**Duyen and Nhon** [33]	*Education*	*If-then manually created rules*		X		X	X	3
**Fast et al.** [49]	*Housing*	*Word2Vec vector generation + cosine distance comparison*	X	X	X	/	X	4.5
**Hao et al.** [41]	*Housing*	*Half-Duplex Search algorithm*		X		X	X	3
**Kim and Chung** [42]	*Health*	*Neural Network*	X		X	/		2.5
**Kim and Chung** [22]	*Health*	*Pearson’s correlation coefficient + Collaborative Filtering* [60,61,62]		X		X	X	3
**Machado et al.** [44]	*Housing*	*Multi-entity Bayesian Networks* [63]		X		/	X	2.5
**Martín-Ruiz et al.** [47]	*Health*	*Rules manually created by experts*		X		X	X	3
**Olszewski and Turek** [26]	*Mobility*	*CART Trees* [64]	/	X	X	X	X	4.5
**Olszewski et al.** [21]	*Government*	*Fuzzy logical rules* [65]		X	/	X	X	3.5
**Orlowski et al.** [25]	*Mobility*	*Fuzzy logical rules*		X	/	X	X	3.5
**Peral et al.** [35]	*Health*	*C4.5 Tree* [66]		X		X	/	2.5
**Persaud et al.** [20]	*Mobility*	*Deep Neural Network*	X		X		/	2.5
**Riboni et al.** [50]	*Health*	*Random Forest* [67]	X	/	X	/	X	4
**Sii-Mobility** [16,17,18]	*Mobility*	*Bayesian Regression ANNs* [68,69,70]	X	/	X	/	X	4
**Xu and Li** [32]	*Risk/Resource Management*	*Fuzzy logical rules*		X	/	X	X	3.5
**Zavala et al.** [30]	*Mobility*	*RIPPER rules* [71]		X		X	X	3
